# Sociodemographic determinants and prevalence of metabolic risk factors and the metabolic syndrome among women aged 20–49 in kirinyaga county, Kenya

**DOI:** 10.3389/fepid.2026.1807836

**Published:** 2026-08-06

**Authors:** Umotho Kinya Mbae, Florence Kyallo, Dorothy Othoo, Zipporah Ndungu, Beatrice N. Kiage Mokua, Vincent Were

**Affiliations:** 1 Jomo Kenyatta University of Agriculture and Technology, Nairobi, Kenya; 2 African Population and Health Research Centre, Nairobi, Kenya

**Keywords:** diabetes mellitus, dyslipidemia, hypertension, metabolic syndrome, noncommunicable diseases, obesity, prevalence, sociodemographic determinants

## Abstract

**Introduction:**

Globally, Noncommunicable diseases (NCDs) contribute to 75% of all deaths. The precursors to the development of NCDs include the metabolic syndrome (MetS) and key metabolic risk factors outlined by the World Health Organisation, including overweight/obesity, raised blood pressure, hyperglycemia, and hyperlipidemia. Women in Kirinyaga County in Kenya have the highest prevalence of overweight and obesity at 64.6%, and hypertension at 20%, as per the 2022 Kenyan Demographic Health survey; however, data on MetS, hyperglycemia, and hyperlipidemia in Kirinyaga is scarce. Therefore, this study aimed to determine the relationship between sociodemographic determinants and the prevalence of metabolic risk factors and the metabolic syndrome among women aged 20–49 years in Kirinyaga County, Kenya.

**Methods:**

A cross-sectional study design was employed, and multi-stage sampling was used to select 425 women aged 20–49 years in Kirinyaga County, Kenya. Data were collected on sociodemographic determinants and anthropometric and biochemical measurements. Data analysis was conducted using STATA version 17, frequencies, proportions and binary logistic regression. A total of 425 women participated in the study.

**Results:**

The prevalence of MetS was 46.4%; 68.87% were obese or overweight; central obesity, measured by waist circumference, was 79.3%, and by waist-hip ratio, 51.06%. Diabetes was at 8.56%, elevated blood pressure at 22.6%, elevated triglycerides at 17.2%, Low HDL-C at 68.6%, and elevated cholesterol at 7.5%. The primary determinants of metabolic health were wealth and age. Increasing age (*p* > 0.05) elevated the odds of having all risk factors for NCDs and metabolic syndrome, except low HDL cholesterol. Women in the highest wealth quintile had increased odds of obesity (AOR = 1.83, *p* = 0.036), elevated triglycerides (AOR = 2.22, *p* = 0.035), and metabolic syndrome (AOR = 1.79, *p* = 0.040). However, being a student reduced the odds of metabolic syndrome (AOR = 0.24, *p* = 0.047). Hormonal contraceptive use, on one hand, was associated with reductions in hypertension (AOR = 0.32, *p* < 0.001), but was a significant risk factor for low HDL cholesterol (AOR = 2.08, *p* = 0.011).

**Conclusion:**

The study reveals a high prevalence of both metabolic syndrome and metabolic risk factors linked to noncommunicable diseases, mainly driven by ageing, multi-parity, and economic affluence. Public health interventions need to focus on wealth-associated lifestyle behaviours.

## 1 Introduction

Noncommunicable diseases (NCDs) are a major public health challenge, contributing to 75% of all deaths worldwide (1), with 73% of these deaths happening in low and middle-income countries. The key metabolic risk factors for NCDs, according to the World Health Organisation, include being overweight or obese, having raised blood pressure, including hypertension, hyperglycemia, and hyperlipidemia (1). The clustering of metabolic risk factors in a single individual usually indicates the presence of the metabolic syndrome (MetS), which, according to the Joint Interim statement, is diagnosed when one has three or more of the following abnormalities: elevated waist circumference, elevated triglycerides, reduced high-density lipoprotein cholesterol, elevated blood pressure or elevated fasting glucose (2).

Globally, 2.5 billion people are overweight, with 890 million affected by obesity, 830 million people are living with diabetes, and 1.4 billion adults between 30 and 79 have hypertension, and only 320 million people have it controlled (3–5). When it comes to dyslipidemias, the global prevalence of hypertriglyceridemia is 28.85, hypercholesterolemia is 24.1%, and low HDL-C is 38.4% (6). In Africa, 18.5%% of women and 8.8% of men are obese, thirty-six per cent are hypertensive, 8.7% of the women and 8.3% of the men have diabetes, and the prevalence of dyslipidemia ranges from 5.2% to 88.9% (7, 8).

When it comes to metabolic syndrome, 1.54 billion adults globally have the metabolic syndrome, with women leading at 31% (846 million), while 25.7% (692 million) of men have MetS (9). In Africa, the prevalence of the metabolic syndrome is currently at 32.4%, with females taking the lead at a prevalence of 36.9%. In comparison, males have a prevalence of 26.7%. Within African regions, South Africa is the most affected at 33.6%, followed by West Africa at 33.1%, North Africa at 32.8%, East Africa at 30.3%, and Central Africa at 30.1% (10).

In Kenya, the prevalence of metabolic risk factors shows a clear gender disparity, where women are disproportionately affected. National demographic data highlights that forty-five per cent of women are either obese or overweight, as compared to seventeen per cent of men (11). Hypertension is at nine per cent among women, while three per cent of the men have been diagnosed with hypertension; the prevalence of elevated blood glucose is at 1% for both men and women (11). Meanwhile, 7.3% of men and 12.8% of women in Kenya have high cholesterol levels (12). In Kenya, the prevalence of the Mets in the urban population was at 25.6% (13). Amongst the 47 counties in Kenya, women in Kirinyaga County have the highest prevalence of obesity or overweight, at 64.6% of the women are either obese or overweight, and the prevalence of hypertension among women is at 20% (11).

The presence of the metabolic risk factors for NCDs has profound long-term consequences if left unaddressed. Apart from the immediate clinical burden, they are major determinants of disability adjusted life years (DALYs) and premature mortality. In 2021 alone, high body mass index (BMI) contributed to 3.7 million deaths from cancers, cardiovascular diseases, neurological disorders, diabetes, digestive diseases and chronic respiratory diseases (14). Furthermore, high blood pressure caused 11 million deaths (5), while high systolic blood pressure led to 255.5 million DALYs. Diabetes and kidney disease, as a result of diabetes, led to over two million deaths in 2021 (4), while high fasting plasma glucose led to 155.7 million Disability Adjusted Life Years (14).

Despite this clear evidence of an obesity and hypertension crisis among women in Kirinyaga County, data regarding the full spectrum of metabolic health, specifically blood glucose levels, lipid profiles and the overall magnitude of the metabolic syndrome, remains scarce. The literature suggests that these metabolic risks are driven by a complex interplay of socio-demographic and socio-economic determinants as risk factors for non-communicable diseases: age, pregnancy, parity, menopause, use of birth control pills, marital status, educational level, residential area, employment status, and household wealth index (13, 15–18).

Consequently, it is important to map out these risk factors to inform comprehensive targeted interventions. Thus, this study aimed to determine the prevalence of key metabolic risk factors and metabolic syndrome among women aged 20–49 years in Kirinyaga County, and to examine the socio-demographic determinants associated with these risks.

## 2 Materials and methods

### 2.1 Study area

The study was conducted in Kirinyaga County. The county is strategically located along the equator on the windward side of Mt. Kenya. It has a tropical climate and an equatorial rainfall pattern, making it suitable for agriculture. Agriculture is the county's main economic activity, accounting for 43.9% of its GDP. It produces some of the world's finest coffee and the most aromatic rice in East Africa. It also boasts agricultural exports, including horticulture and macadamia. The county is divided into five sub-counties: Kirinyaga East, Kirinyaga West, Mwea East, Mwea West, and Kirinyaga Central. Kirinyaga County has an estimated population of 610,411, comprising 49.4% males and 50% females. 6% females, as per the 2019 Kenya Population and Housing Census. It lists the number of females aged 20–49 years to be a total of 134,219, where Kirinyaga Central has a total of 27,181 women, Kirinyaga East has 26,605 women, Kirinyaga West has 24,417 women, Mwea East (Kirinyaga South) has 30,661 women, Mwea East (Kirinyaga North) has 23,339 women, and Mt. Kenya Forest has 15 women.

Kirinyaga County has one Public Level Five hospital, known as Kerugoya County Referral, located in Kirinyaga Central. It has three Level Four public hospitals in Kirinyaga South, Kirinyaga East, and Kirinyaga West. It has 23 public Level Three healthcare facilities and 45 public Level Two healthcare facilities. This study was conducted across two sub-counties: Kirinyaga North (Mwea West) and Kirinyaga Central.

### 2.2 Study design

A cross-sectional study was used to collect data on sociodemographic and metabolic risk factors associated with noncommunicable diseases, and data were collected in July 2025. The merits of the cross-sectional study design include the fact that exposure and outcome are measured simultaneously, allowing data to be collected once and then analysed.

### 2.3 Study population

The study population consisted of females of reproductive age (20–49 years) who were residents of Kirinyaga County for the past year (19). Females who were residents of Kirinyaga County but were expectant, or three months postpartum, were excluded, as they could affect the study estimates (15).

### 2.4 Sample size determination and the sampling procedure

The sample size of 423 persons was determined using the formula by Fisher et al. (74), shown below

$$n=\frac{Z^{2}*p(1-p)}{e^{2}}$$

Where *n* = desired sample size (if the population is more than 10,000).

z = standard normal deviate at the required confidence level (Set at 1.96, corresponding to 95%).

*p* = population proportion estimated to have metabolic syndrome, set at 50% because it is unknown.

d = desired precision set at 0.05.

Therefore, on substitution:

*n* = (1.96 × 1.96 × 0.5 × 0.5) ÷ (0.05 × 0.05)

= (0.9604) ÷ (0.0025)

= 384.16

To account for attrition, a 10% non-response rate was added.

*n* = 384.16 + (10/100) 384.16

= 422.56 ≈ 423

Multi-stage random sampling methods were used to select the study participants. Kirinyaga County has five sub-counties.

Two of the sub-counties were randomly selected, representing 1/3 of the five sub-counties. The two sub-counties had a total of 9 wards, and 1/3 of these wards were randomly selected to participate in the study. Sampling was then conducted in community units within these wards (Supplementary File One Included).

### 2.5 Data collection procedures

Demographic information was collected through a structured questionnaire, administered by the researcher. It collected data on individuals, including age, marital status, gravidity and parity, family planning education, occupation, and housing and social amenities.

Physical measurements:

- Weight was measured using a calibrated SECA weighing scale, and two measurements were taken for each person. Weight was measured to the nearest 0.1 kg. Participants were asked to remove heavy outer garments, shoes, and accessories. They then stood still on the centre platform, with their weight evenly distributed on both feet and their arms hanging loosely at their sides.
- Height was measured using an adult stadiometer, and two measures were taken for each person. It was measured to the nearest 0.1 cm using a calibrated stadiometer after participants removed their shoes and headgear. Participants stood upright, with their heels, buttocks, and scapulae in contact with the vertical board. The movable headpiece was then lowered to gently compress the hair and rest firmly on the highest point of the head.
- Waist and hip circumferences were measured with a SECA non-stretchable waist tape to the nearest 0.1 cm, with two measurements taken with each tape. Waist circumference measurement was taken at the midpoint between the inferior margin of the last palpable rib and the top of the iliac crest. The tape was kept horizontal to the floor, and the reading was recorded at the end of a normal expiration without compressing the skin. Hip circumference measurement was taken at the level of the maximum posterior extension of the buttocks.
- Blood pressure was measured using an OMRON Automatic blood pressure monitor, with an appropriately sized cuff based on the participant's upper arm circumference. Prior to the measurements, participants rested for at least 5 min in a seated position. The measurement was conducted on the left arm while the participant was seated with back support, with the arm supported on a table at heart level. Three consecutive readings were taken.

Biochemical measurements: To avoid pricking patients twice, an expert phlebotomist drew blood from each patient once, using the antecubital vein. A drop of whole blood from the syringe was used to estimate blood glucose levels using the On-Call Plus glucometer. The remaining blood was centrifuged at approximately 3,000 rpm for 10–15 min to separate plasma from blood cells. Since the analysis could not be performed at the collection point. The plasma fraction was transported to Kerugoya County Referral Hospital, where it was used to determine the lipid profile.

### 2.6 Pre-testing

The tools were pre-tested in Inoi ward with 42 participants, representing 10% of the total sample, and the results helped refine and standardise the questionnaire. Statistical reliability was determined using Cronbach's alpha, which was 70% and thus considered satisfactory.

### 2.7 Data management and analysis

Data collection was done through the Commcare app. The data were then downloaded and exported to STATA version 17for data analysis. The data was analysed using descriptive and inferential statistics. Frequencies and proportions were used to analyse sociodemographic data and the prevalence of metabolic risk factors for noncommunicable diseases and the metabolic syndrome. Binary logistic regression was employed to determine the relationships between sociodemographic factors and metabolic risk factors for noncommunicable diseases, as well as between sociodemographic factors and metabolic syndrome.Statistical significance was determined at the 95% confidence level, with a *p*-value threshold of <0.05.

The wealth quintiles were computed using the Multiple Correspondence Analysis (20). It created a composite socio-economic status (SES) index based on household assets and characteristics. The following households’ assets and characteristics were used to calculate it: dwelling type, housing rental status, toilet facility, and access to electricity, water source, and cooking energy used by the household. The wealth quintiles were then divided into three categories: low, middle, and high.

In the study, metabolic risk factors and the metabolic syndrome were defined as follows: overweight was defined as a BMI above 25 kg/m2, and obesity was defined as a BMI greater than or equal to 30 kg/m2 (21). Abdominal obesity was defined as either a waist circumference of 80 cm (20) or greater or a waist-to-hip ratio greater than 0.85 (22). Pre-diabetes was defined as fasting blood glucose 5.6–6.9 mmol/L, while diabetes was defined as fasting blood glucose > 6.9 mmol/L (23). Elevated blood pressure was defined as an average of three readings, and women with systolic BP equal to or above 130 mmHg or diastolic BP equal to or above 85 mmHg were categorised as hypertensive (2). Dyslipidemia was defined by an abnormality in any of the following three lipid parameters. The parameters included triglyceride levels greater than or equal to 1.7 mmol/L, HDL-C levels equal to or lower than 1.3 mmol/L, and cholesterol levels above 5 mmol/L (2). Metabolic syndrome was defined according to the 2009 Joint Interim Statement (2), where presence of three or more of the following risk factors indicated the presence of MetS; a waist circumference greater than or equal to 80 cm; fasting blood glucose that was greater than or equal to 5.6 mmol/L; elevated blood pressure, where the systolic blood pressure was greater than or equal to 130mmhg and the diastolic blood pressure was greater than or equal to 85mmhg; High-density lipoprotein below 1.3 mmol/L and triglycerides greater than or equal to 1.7 mmol.

### 2.8 Ethical considerations

The study strictly adhered to the Helsinki declarations. The JKUAT Institutional Scientific and Ethics Review Committee (ISEREC) reviewed the study and granted ethical approval (License no.). JKU/ISERC/02316/1727. Ethical permission was also granted by the National Commission for Science, Technology, and Innovation (NACOSTI), Kenya license no: NACOSTI/P/25/4174059. Each Study participant confirmed their willingness to participate by signing an informed consent form.

## 3 Results

A 100.5% response rate was achieved, with 425 participants from Kirinyaga County participating in the research study.

Table 1 details the sociodemographic and reproductive characteristics of the study participants. The majority of the women were between 30 and 49 years old. The age groups with the highest numbers of participants were 40–49 (39.1%) and 30–39 (37.9%). Most women came from households with fewer than 4 persons below 18 years (96.24%) and fewer than 4 persons above 18 years (87.06%). Regarding marital status (71.3%) were currently married. Regarding reproductive history, the vast majority of women reported a parity of 4 or fewer (95.5%) and a gravidity of 4 or fewer (94.4%). Contraceptive use was high, where 27.53% did not use it, and 72.46 of the women used it; hormonal contraception was used by 59.8% of the participants.

**Table 1:** Socio-demographic characteristics of women aged 20–49 years in kirinyaga county, Kenya (*N* = 425). A 100.5% response rate was achieved with 425 participants from Kirinyaga participating in the research study.

Variable	Percentages (%)	Frequency (n)
Age (years)		
20–29	23.06	98
30–39	37.88	161
40–49	39.06	166
Household members under 18		
Below 4 persons	96.24	409
4 persons and above	3.76	16
Household members over 18		
Below 4 persons	87.06	370
4 persons and above	12.94	55
Marital status		
Never married	10.82	46
Currently married	71.29	303
Separated	12.24	52
Divorced	2.12	9
Widowed	2.82	12
Cohabitation	0.71	3
Gravidity		
≤4 pregnancies	94.35	401
>4 pregnancies	5.65	24
Parity		
≤4 births	95.53	406
>4 births	4.47	19
Family planning use (FP use)		
None	27.53	117
Non-hormonal	2.35	10
Hormonal	59.76	254
Intrauterine Devices	10.35	44

Categorical variables are reported as frequency (n) and percentage (%).

Table 2 summarises the socioeconomic characteristics of the study participants (*N* = 425). The majority of participants (86.0%) had completed at least secondary education, with 41.2% holding a college, university, or postgraduate degree. Regarding employment, the largest proportion of participants were self-employed (39.8%) or working in the non-government sector (35.5%). A significant segment of the study population consisted of students (18.6%). Economically, participants were distributed across the three wealth quintiles: Low (33.4%), Middle (33.7%), and High (32.9%). The reported individual earnings revealed that 26.35% of the women earned below KES 3,000, 27.53% earned between KES 3,000–7,000, 30.36% earned between KES 7,000–15,000, and 15.76% earned above KES 15,000.

**Table 2:** Social economic characteristics of women aged 20–49 years in kirinyaga county (*N* = 425).

Variable	Percentages (%)	frequency (n)
Highest education		
No formal schooling	0.94	3
Primary completed	13.18	56
Secondary completed	30.82	131
High school completed	13.88	59
College/University completed	26.82	114
Postgraduate degree	14.35	61
Employment		
Government employee	3.29	14
Non-government employee	35.53	151
Self-employed	39.76	169
Student	18.59	79
Informal Employment	2.82	12
Wealth Quintile		
Low	33.41	142
Middle	33.65	143
High	32.94	140
Monthly income (KES)		
Below 3,000	26.35	112
3,000–7,000	27.53	117
7,000–15,000	30.36	129
Above 15,000	15.76	67

KES, kenyan shilling. Categorical variables are presented as frequency (*n*) and percentage (%).

Table 3 presents the prevalence of the metabolic risk factors. The results show that 68.87% of the women in Kirinyaga County were either obese or overweight. The results show that the prevalence of abdominal obesity, as measured by waist circumference, was 79.3% and, when categorised by waist-to-hip ratio, 51.06%. The prevalence of abdominal obesity was 51.06%. Glycemic indicators among fasting participants (*n* = 362) show that the prevalence of pre-diabetes in Kirinyaga County was 55.25%, while the prevalence of diabetes was 8.56%. The cardiovascular markers indicate that the prevalence of elevated blood pressure among women aged 20–49 years in Kirinyaga County was 22.6%. The results show that 17.2% of the women had elevated triglycerides, 68.6% had low high-density lipoprotein levels, and 7.5% had elevated cholesterol levels.

**Table 3:** The prevalence of the metabolic risk factors for non-communicable diseases among women aged 20–49 in kirinyaga county, Kenya.

Indicator	Frequency	Percentage
BMI Category *n* = 425		
Underweight (>18.5 kg/m^2^)	14	3.30
Normal (18.5 kg/m^2^–24.9 kg/m^2^)	118	27.83
Overweight (25 kg/m^2^–29.9 kg/m^2^)	124	29.25
Obese (≥ 30 kg/m^2^)	168	39.62
Waist Circumference *n* = 425		
Normal (<80 cm)	88	20.7
Abdominal obesity (≥ 80 cm)	337	79.3
Waist-Hip Ratio		
Normal (≤ 0.85)	208	48.94
Abdominal obesity (> 0.85)	217	51.06
Blood Glucose Levels *n* = 362^a^		
Normal (<5.6 mmol/L)	131	36.19
Pre-diabetes (≥5.6 mmol/L & ≤ 6.9 mmols)	200	55.25
Diabetic (> 6.9 mmol/L)	31	8.56
Blood Pressure *n* = 425		
Normal BP (≤ 130/85)	329	77.40
High BP (≥130/85)	96	22.60
Dyslipidemia *n* = 425		
Triglycerides		
Normal (<1.7 mmol/L)	352	82.80
Elevated (≥1.7 mmol/L)	73	17.20
HDL cholesterol		
Normal (≥1.3 mmol/L)	124	31.40
Low (<1.3 mmol/L)	271	68.60
Cholesterol		
Normal (≤5 mmol/L)	393	92.50
Elevated (>5 mmol/L)	32	7.50

a Analysis restricted to participants who fasted (*n* = 362).

BMI, body mass index.

HDL, high density lipoprotein.

BP, blood pressure.

Table 4 shows that the prevalence of the metabolic syndrome in Kirinyaga County was 46.4% (*n* = 197). This indicates that nearly half of the women aged 20–49 years in Kirinyaga County presented with a clustering of at least three cardiovascular risk factors. The remaining 53.6% (*n* = 228) had two or fewer risk factors and were classified as not having the syndrome.

**Table 4:** Prevalence of the metabolic syndrome among women aged 20–49 in kirinyaga county (*N* = 425).

Metabolic Syndrome Status	N	%
No Metabolic Syndrome (< 3 risk factors)	228	53.6
Metabolic Syndrome (≥ 3 risk factors)	197	46.4
Total	425	100

Metabolic syndrome was defined as the presence of three or more of the following risk factors.

A waist circumference greater than or equal to 80 cm.

Fasting blood glucose that was greater than or equal to 5.6 mmol/L.

Elevated blood pressure, where the systolic blood pressure was greater than or equal to 130mmhg and the diastolic blood pressure was greater than or equal to 85mmhg.

High-density lipoprotein below 1.3 mmol/L and.

Triglycerides greater than or equal to 1.7 mmol.

Table 5 presents the factors associated with obesity (BMI ≥ 25 kg/m^2^). The results show that increasing age was significantly associated with higher odds of obesity, at a crude OR of 1.04 (95% CI: 1.01 −1.07, *p* = 0.003), and an adjusted OR of 1.04 (95% CI: 1.00–1.09, *p* = 0.026). Parity was also associated with a higher odds of being obese, with a crude OR of 1.21 (95% CI: 1.02–1.44, *p* = 0.028). Women who were also separated had higher odds of being obese, this was at Crude OR of 2.56 (95% CI: 1.07–6.12, *p* = 0.034). Regarding economic status, women who were informally employed also had lower odds of obesity, with a crude OR of 0.09 (95% CI: 0.01–0.56, *p* = 0.01). Women in the high and middle income wealth quintiles were at higher odds of being obese, for women in the middle income wealth quintiles, this was at a Crude OR of 1.84 (95% CI: 1.12–3.03 *p* = 0.016) and at an Adjusted OR of 1.84 (95% CI: 1.08–3.13 *p* = 0.025) and for women in the high income wealth quintiles at a Crude OR 2.07 (95%CI: 1.25–3.44 and *p* = 0.005) at an Adjusted OR 2.43 (95% CI: 1.33–4.47 *p* = 0.004).

**Table 5:** Determinants of obesity (BMI ≥ 25 kg/m^2^) among women aged 20–49 in kirinyaga county, Kenya (*N* *=* *425*).

Variable	Crude OR (95% CI)	*p*-value	AOR (95% CI)	*p*-value
Age (years)	1.04 (1.01–1.07)	0.003*	1.04 (1.00–1.09)	0.026*
Gravidity	1.12 (0.96–1.32)	0.157	0.74 (0.49–1.12)	0.157
Parity	1.21 (1.02–1.44)	0.028*	1.37 (0.86–2.17)	0.189
Years of schooling	0.98 (0.92–1.06)	0.667	1.08 (0.90–1.29)	0.409
Marital status				
Never married	Reference	–	Reference	–
Currently married	1.74 (0.92–3.27)	0.087	0.95 (0.41–2.21)	0.901
Separated	2.56 (1.07–6.12)	0.034*	1.34 (0.48–3.74)	0.576
Divorced	2.69 (0.50–14.39)	0.247	1.54 (0.25–9.56)	0.643
Widowed	1.54 (0.41–5.84)	0.527	0.66 (0.15–2.93)	0.586
Cohabitating	1.54 (0.13–18.19)	0.733	0.59 (0.04–8.99)	0.701
Family planning use				
None	Reference	–	Reference	–
Hormonal	1.15 (0.72–1.83)	0.554	1.22 (0.64–3.25)	0.496
Non-hormonal	4.68 (0.57–38.2)	0.150	2.78 (0.32–24.24)	0.355
Intra-Uterine Devices	1.39 (0.66–2.98)	0.404	1.20 (0.52–2.75)	0.668
Highest education				
Primary incomplete	Reference	–	Reference	–
Primary completed	1.53 (0.80–2.93)	0.201	1.27 (0.57–2.82)	0.564
Secondary incomplete	2.05 (0.91–4.60)	0.082	1.95 (0.58–6.53)	0.280
Secondary completed	1.16 (0.60–2.23)	0.66	0.85 (0.22–3.24)	0.815
College/University completed	0.96 (0.46–2.00)	0.906	0.55 (0.10–2.91)	0.482
Employment				
Government employee	Reference	–	Reference	–
Non-government employee	0.76 (0.20–2.85)	0.681	0.74 (0.18–3.16)	0.704
Self-employed	0.63 (0.17–2.36)	0.494	0.89 (0.20–3.86)	0.880
Student	0.47 (0.12–1.82)	0.275	0.52 (0.11–2.34)	0.395
Informal employment	0.09 (0.01–0.56)	0.01*	0.19 (0.03–1.40)	0.104
Average income	1.00 (1.00–1.00)	0.19	1.00 (1.00–1.00)	0.632
Wealth quintile				
Low	Reference	–	Reference	–
Middle	1.84 (1.12–3.03)	0.016*	1.84 (1.08–3.13)	0.025*
High	2.07 (1.25–3.44)	0.005*	2.43 (1.33–4.47)	0.004*

OR, odds ratio; CI, confidence interval; AOR, adjusted odds ratio.

* *p* ≤ 0.05.

Table 6 details the factors associated with abdominal obesity (waist circumference ≥ 80 cm). The results of the study show that in the bivariate analysis increases in age, gravidity, and parity were associated with greater odds of developing of abdominal obesity, at a Crude ORs of 1.03 (95% CI: 1.00–1.05 *p* = 0.022), 1.29 (95% CI: 1.10–1.50 *p* = 0.001), and 1.38 (95% CI: 1.17–1.63 *p* = 0), respectively. While an increase in years of schooling, with a crude OR of 0.94 (95% CI: 0.88–1.00, *p* = 0.06), reduced the odds of developing abdominal obesity. Women who also used intra-uterine methods of family planning had increased odds of having abdominal obesity at a Crude OR of 2.18 (95% CI: 1.06–4.48, *p* = 0.034). However, after adjusting for potential confounders (AOR), the only variable that remained a significant predictor of abdominal obesity in the multivariate model was wealth, with women in the high wealth quintiles having nearly double the odds of abdominal obesity compared to those in the low wealth quintiles at an adjusted OR 1.83 (95% CI: 1.04–3.23, *p* = 0.036).

**Table 6:** Determinants of abdominal obesity (waist circumference ≥80 cm) among women aged 20–49 in kirinyaga county, Kenya (*N* = 425).

Variable	Crude OR (95% CI)	*p*-value	AOR (95% CI)	*p*-value
Age (years)	1.03 (1.00–1.05)	0.022*	1.01 (0.97–1.04)	0.704
Gravidity	1.29 (1.10–1.50)	0.001*	0.90 (0.61–1.33)	0.554
Parity	1.38 (1.17–1.63)	0*	1.49 (0.96–2.31)	0.076
Years of schooling	0.94 (0.88–1.00)	0.06*	1.15 (0.97–1.37)	0.106
Marital status				
Never married	Reference	–	Reference	–
Currently married	1.57 (0.84–2.94)	0.16	0.82 (0.36–1.83)	0.640
Separated	1.32 (0.59–2.93)	0.501	0.78 (0.31–2.01)	0.614
Divorced	2.84 (0.63–12.80)	0.174	1.81 (0.35–9.37)	0.482
Widowed	1.99 (0.55–7.22)	0.296	0.74 (0.18–3.08)	0.683
Cohabitating	0.71 (0.06–8.41)	0.786	0.30 (0.02–4.66)	0.391
Family planning use				
None	Reference	–	Reference	–
Hormonal	1.18 (0.76–1.83)	0.455	1.02 (0.60–1.71)	0.392
Non-hormonal	0.48 (0.12–1.96)	0.309	0.27 (0.06–1.21)	0.088
Intra-Uterine Devices	2.18 (1.06–4.48)	0.034*	1.71 (0.78–3.74)	0.179
Highest education				
Primary incomplete	Reference	–	Reference	–
Primary completed	1.33 (0.72–2.47)	0.358	1.09 (0.52–2.30)	0.819
Secondary incomplete	1.82 (0.87–3.82)	0.111	1.27 (0.42–3.87)	0.671
Secondary completed	0.61 (0.32–1.15)	0.124	0.33 (0.09–1.16)	0.085
College/University completed	0.65 (0.32–1.33)	0.240	0.28 (0.06–1.38)	0.118
Employment				
Government employee	Reference	–	Reference	–
Non-government employee	0.67 (0.22–2.01)	0.471	0.55 (0.16–1.86)	0.335
Self-employed	0.88 (0.29–2.63)	0.812	0.80 (0.23–2.86)	0.737
Student	0.94 (0.30–2.97)	0.92	0.73 (0.20–2.73)	0.645
Informal employment	0.25 (0.05–1.34)	0.106	0.36 (0.06–2.24)	0.271
Average income	1.00 (1.00–1.00)	0.69	1.00 (1.00–1.00)	0.781
Wealth quintile				
Low	Reference	–	Reference	–
Middle	1.17 (0.73–1.86)	0.515	1.35 (0.82–2.23)	0.244
High	1.26 (0.79–2.00)	0.34	1.83 (1.04–3.23)	0.036*

OR, odds ratio; CI, confidence interval; AOR, adjusted odds ratio.

* *p* ≤ 0.05.

Table 7 examines the determinants of elevated blood pressure (BP > 130/85 mmHg). The results of this study revealed that increasing age increased the odds of becoming hypertensive, with a Crude OR of 1.10 (95% CI: 1.06 –1.14, *p* < 0.001) and an Adjusted OR of 1.09 (95% CI: 1.05–1.14, *p* < 0.001). Gravidity and parity also increased the odds of developing hypertension, with Crude ORs of 1.28 (95% CI: 1.08–1.53, *p* = 0.005) and 1.32 (95% CI: 1.09–1.59, *p* = 0.004), respectively. On the other hand, the years of schooling reduced the odds of developing hypertension, with a Crude OR of 0.84 (95% CI: 0.77–0.91, *p* < 0.001). Persons with secondary and tertiary education had lower odds of becoming hypertensive, with Crude ORs of 0.58 (95% CI: 0.35–0.94, *p* = 0.026) and 0.21 (95% CI: 0.08–0.55, *p* = 0.002), respectively. When it came to marital status, women who were in a union had higher odds of developing hypertension, this was at a Crude OR 3.33 (95% CI: 1.16–9.61, *p* = 0.026). Regarding family planning, women who used hormonal methods had lower odds of developing hypertension, with a Crude OR of 0.34 (95% CI: 0.20–0.56, *p* < 0.001) and an adjusted OR of 0.32 (95% CI: 0.18–0.59, *p* < 0.001).

**Table 7:** Determinants of elevated blood pressure (BP > 130/85 mmHg) among women aged 20–49 in kirinyaga county, Kenya (*N*-425).

Variable	Crude OR (95% CI)	*p*-value	Adjusted OR (95% CI)	*p*-value
Age (years)	1.10 (1.06–1.14)	<0.001*	1.09 (1.05–1.14)	<0.001*
Gravidity	1.28 (1.08–1.53)	0.005*	1.03 (0.66–1.61)	0.884
Parity	1.32 (1.09–1.59)	0.004*	0.91 (0.56–1.48)	0.715
Years of schooling	0.84 (0.77–0.91)	<0.001*	0.86 (0.73–1.02)	0.078
Marital status				
Never married	Reference	–	Reference	–
Currently married/Cohabiting	3.33 (1.16–9.61)	0.026*	1.53 (0.60–7.82)	0.241
Separated/Divorced/Widowed	3.15 (0.94–10.58)	0.063	1.31 (0.34–5.06)	0.696
Family planning use				
None	Reference	–	Reference	–
Hormonal	0.34 (0.20–0.56)	<0.001*	0.32 (0.18–0.59)	<0.001*
Non-hormonal	0.79 (0.19–3.24)	0.748	0.49 (0.11–2.26)	0.300
Intra-Uterine Devices	0.78 (0.37–1.65)	0.511	0.54 (0.23–1.24)	0.147
Education				
Primary level	Reference	–	Reference	–
Secondary level	0.58 (0.35–0.94)	0.026*	1.53 (0.64–3.65)	0.335
College/University	0.21 (0.08–0.55)	0.002*	0.61 (0.14–2.73)	0.519
Employment				
Government employee	Reference	–	Reference	–
Non-government employee	1.07 (0.28–4.04)	0.926	0.54 (0.12–2.44)	0.417
Self-employed	1.17 (0.31–4.41)	0.812	0.57 (0.12–2.63)	0.468
Student	1.01 (0.25–4.02)	0.994	0.50 (0.10–2.47)	0.399
Informal employment	0.33 (0.03–3.72)	0.372	0.73 (0.05–10.30)	0.813
Average income	1.00 (1.00–1.00)	0.418	1.00 (1.00–1.00)	0.921
Wealth quintile				
Low	Reference	–	Reference	–
Middle	1.11 (0.64–1.93)	0.699	1.35 (0.73–2.50)	0.346
High	0.90 (0.51–1.58)	0.71	1.58 (0.82–3.22)	0.203

OR, odds ratio; CI, confidence interval; AOR, adjusted odds ratio.

* *p* ≤ 0.05.

Table 8 shows the relationship between elevated fasting blood glucose (≥ 5.6 mmol/L) and socio-demographic factors. Increasing age was associated with higher odds of having elevated blood glucose, with a crude OR of 1.05 (95% CI: 1.03–1.08, *p* < 0.001). Regarding schooling, individuals who had not completed secondary education and those who had completed it had lower odds of having elevated blood glucose, with crude ORs of 0.32 (95% CI: 0.15–0.70, *p* = 0.004) and 0.49 (95% CI: 0.25–0.99, *p* = 0.046), respectively. Regarding employment, students had lower odds of developing elevated fasting blood glucose levels, with a crude OR of 0.15 (95% CI: 0.03–0.74, *p* = 0.019). Women who used hormonal family planning also had a lower odds of elevated blood glucose, with a crude OR of 0.41 (0.25–0.66, *p* < 0.001) and an adjusted OR of 0.47 (0.27–0.82, *p* = 0.009).

**Table 8:** Determinants of elevated fasting blood glucose among women aged 20–49 in kirinyaga county, Kenya (*N* = 362).

Variable	Crude OR (95% CI)	*p*-value	Adjusted OR (95% CI)	*p*-value
Age (years)	1.05 (1.03–1.08)	<0.001*	1.03 (0.99–1.07)	0.125
Gravidity	1.13 (0.97–1.32)	0.121	0.82 (0.55–1.22)	0.328
Parity	1.17 (0.99–1.38)	0.065	1.29 (0.83–2.01)	0.263
Years of schooling	0.95 (0.89–1.02)	0.157	1.01 (0.84–1.21)	0.893
Marital status				
Never married	Reference	–	Reference	–
Currently married/Cohabiting	1.02 (0.54–1.93)	0.941	1.11 (0.48–2.55)	0.807
Separated/Divorced/Widowed	1.96 (0.89–4.35)	0.097	1.37 (0.53–3.56)	0.513
Family planning use				
None	Reference	–	Reference	–
Hormonal	0.41 (0.25–0.66)	<0.001*	0.47 (0.27–0.82)	0.009*
Non-hormonal	2.83 (0.34–23.34)	0.333	2.15 (0.25–18.81)	0.489
Intra-Uterine Devices	0.67 (0.341–1.45)	0.311	0.58 (0.26–1.33)	0.200
Education				
Primary incomplete	Reference	–	Reference	–
Primary completed	0.68 (0.34–1.36)	0.276	0.73 (0.32–1.67)	0.461
Secondary incomplete	0.32 (0.15–0.70)	0.004*	0.48 (0.15–1.54)	0.218
Secondary complete	0.49 (0.25–0.99)	0.046*	0.64 (0.17–2.44)	0.519
College/University	0.68 (0.31–1.51)	0.346	0.78 (0.15–4.19)	0.777
Employment				
Government employee	Reference	–	Reference	–
Non-government employee	0.32 (0.07–1.47)	0.143	0.36 (0.07–1.83)	0.217
Self-employed	0.36 (0.08–1.69)	0.197	0.41 (0.08–2.15)	0.291
Student	0.15 (0.03–0.74)	0.019*	0.20 (0.04–1.07)	0.060
Informal employment	0.17 (0.03–1.09)	0.061	0.25 (0.03–1.91)	0.182
Average income	1.00 (1.00–1.00)	0.986	1.00 (1.00–1.00)	0.575
Wealth quintile				
Low	Reference	–	Reference	–
Middle	0.64 (0.40–1.04)	0.073	0.65 (0.39–1.10)	0.112
High	0.86 (0.52–1.41)	0.556	1.14 (0.64–2.03)	0.665

OR, odds ratio; CI, confidence interval; AOR, adjusted odds ratio.

* *p* ≤ 0.05.

Table 9 presents the determinants of low HDL cholesterol levels (<1.3 mmol/L). Increasing age appeared protective, with a 3% reduction in the odds of low HDL per year of age, yielding a crude OR of 0.97 (0.94–0.99; *p* = 0.012). It is also interesting to note that women who were separated or divorced were less likely to have low HDL. For separated women, this was associated with a crude OR of 0.26 (95% CI: 0.10–0.66, *p* = 0.004) and an adjusted OR of 0.19 (95% CI: 0.06–0.61, *p* = 0.005). For women who were divorced, the crude OR was 0.16 (95% CI: 0.03–0.74) and the adjusted OR was 0.12 (95% CI: 0.02–0.72). Conversely, the use of family planning methods increased the risk of having low high-density lipoprotein levels. For women using hormonal methods, this was at a crude OR of 2.25 (95% CI: 1.37–6.78 *p* = 0.001) and at an Adjusted OR of 2.08 (95% CI: 1.18–3.65 *p* = 0.011), while for women using the intra-uterine devices, this was at a Crude OR of 2.75 (95%CI: 1.19–6.36 *p* = 0.018) and an Adjusted OR of 2.60 (95% CI: 1.33–6.45 *p* = 0.039).

**Table 9:** Determinants of Low HDL-cholesterol (<1.3 mmol/L) among women aged 20–49 in kirinyaga county (*N* = 425).

Variable	Crude OR (95% CI)	*p*-value	Adjusted OR (95% CI)	*p*-value
Age (years)	0.97 (0.94–0.99)	0.012*	1.00 (0.96–1.04)	0.955
Gravidity	0.90 (0.77–1.06)	0.214	1.08 (0.68–1.72)	0.737
Parity	0.88 (0.74–1.05)	0.147	0.84 (0.51–1.39)	0.502
Years of schooling	1.04 (0.97–1.12)	0.316	0.95 (0.78–1.16)	0.642
Marital status				
Never married	Reference	–	Reference	–
Currently married	0.85 (0.40–1.81)	0.670	0.53 (0.19–1.48)	0.225
Separated	0.26 (0.10–0.66)	0.004*	0.19 (0.06–0.61)	0.005*
Divorced	0.16 (0.03–0.74)	0.019*	0.12 (0.02–0.72)	0.020*
Widowed	0.25 (0.06–1.11)	0.069	0.22 (0.04–1.21)	0.082
Cohabitating	0.63 (0.05–7.64)	0.713	0.22 (0.01–3.22)	0.268
Family planning use				
None	Reference	–	Reference	–
Hormonal	2.25 (1.37–6.78)	0.001*	2.08 (1.18–3.65)	0.011*
Non-hormonal	1.92 (0.47–7.85)	0.362	2.78 (0.62–12.52)	0.183
Intra-Uterine Devices	2.75 (1.19–6.36)	0.018*	2.60 (1.33–6.45)	0.039*
Education				
Primary incomplete	Reference	–	Reference	–
Primary completed	0.55 (0.28–1.09)	0.085	0.52 (0.22–1.23)	0.136
Secondary incomplete	1.67 (0.70–4.02)	0.250	1.71 (0.46–6.38)	0.425
Secondary complete	1.19 (0.57–2.45)	0.646	1.37 (0.31–5.95)	0.676
College/University	0.74 (0.33–1.65)	0.457	0.75 (0.09–3.64)	0.759
Employment				
Government employee	Reference	–	Reference	–
Non-government employee	1.01 (0.32–3.18)	0.988	1.11 (0.31–3.96)	0.871
Self-employed	1.30 (0.41–4.09)	0.653	1.71 (0.45–6.46)	0.429
Student	1.64 (0.49–5.50)	0.425	1.51 (0.38–6.03)	0.556
Other employment	0.97 (0.19–5.03)	0.973	0.58 (0.09–3.64)	0.558
Average income	1.00 (1.00–1.00)	0.861	1.00 (1.00–1.00)	0.950
Wealth quintile				
Low	Reference	–	Reference	–
Middle	1.06 (0.63–1.76)	0.830	1.07 (0.61–1.87)	0.816
High	1.43 (0.85–2.43)	0.180	1.53 (0.81–2.89)	0.185

OR, odds ratio; CI, confidence interval; AOR, adjusted odds ratio.

* *p* ≤ 0.05.

Table 10 presents the determinants of elevated total cholesterol (>5.0 mmol/L). The results show that increasing age, reduced the odds of having elevated cholesterol level at a Crude OR of 0.97 and at (95% CI: 0.94–0.99 *p* = 0.012) but the odds increased at an Adjusted OR 1.07 (95% CI: 1.01–1.15 *p* = 0.029), while women who were previously in a union, had reduced odds of having elevated cholesterol, this was at a Crude OR of 0.26 (95% CI: 0.10–0.66 *p* = 0.004).

**Table 10:** Determinants of elevated total cholesterol (>5.0 mmol/L) among women aged 20–49 in kirinyaga county, Kenya (*N* = 425).

Variable	Crude OR (95% CI)	*p*-value	Adjusted OR (95% CI)	*p*-value
Age (years)	0.97 (0.94–0.99)	0.012*	1.07 (1.01–1.15)	0.029*
Gravidity	0.90 (0.77–1.06)	0.214	0.95 (0.44–2.07)	0.904
Parity	0.88 (0.74–1.05)	0.147	0.75 (0.33–1.70)	0.496
Years of schooling	1.04 (0.97–1.12)	0.316	0.93 (0.69–1.25)	0.647
Marital status				
Never married	Reference	–	Reference	–
Currently married/Cohabiting	0.85 (0.40–1.81)	0.67	6.66 (0.63–70.40)	0.115
Separated/ Divorced/ Widowed	0.26 (0.10–0.66)	0.004*	5.92 (0.52 –66.75)	0.151
Family planning use				
None	Reference	–	Reference	–
Hormonal	0.47 (0.21–1.07)	0.072	0.59 (1.33–7.47)	0.275
Non-hormonal	–	–	–	–
Intra-Uterine Devices	1.66 (0.61–4.52)	0.325	1.79 (0.57–5.57)	0.316
Education				
Primary incomplete	Reference	–	Reference	–
Primary completed	0.55 (0.28–1.09)	0.085	0.88 (0.22–3.52)	0.144
Secondary incomplete	1.67 (0.70–4.02)	0.25	2.42 (0.34–17.15)	0.408
Secondary complete	1.19 (0.57–2.45)	0.646	2.84 (0.31–26.24)	0.676
College/University	0.74 (0.33–1.65)	0.457	2.15 (0.12–36.58)	0.595
Employment				
Government employee	Reference	–	Reference	–
Non-government employee	1.01 (0.32–3.18)	0.988	0.15 (0.01–1.87)	0.140
Self-employed	1.30 (0.41–4.09)	0.653	0.33 (0.03–3.90)	0.382
Student	1.64 (0.49–5.50)	0.425	0.26 (0.02–3.53)	0.313
Other employment	0.97 (0.19–5.03)	0.973	–	–
Average income	1.00 (1.00–1.00)	0.861	1.00 (1.00–1.00)	0.920
Wealth quintile				
Low	Reference	–	Reference	–
Middle	1.06 (0.63–1.76)	0.83	1.13 (0.45–2.83)	0.798
High	1.43 (0.85–2.43)	0.18	1.13 (0.38–3.33)	0.829

OR, odds ratio; CI, confidence interval; AOR, adjusted odds ratio.

* *p* ≤ 0.05.

Table 11 examines the determinants of elevated triglyceride levels (≥ 1.7 mmol/L). The results show that increasing age, increased the odds of having elevated triglycerides, this was at a Crude OR of 1.05 (95% CI: 1.01–1.08 *p* = 0.007) and at Adjusted OR 1.06 (95% CI: 1.01–1.11 *p* = 0.020), while persons in the high wealth quintile had increased odds of having elevated triglycerides, this was at an Adjusted OR of 2.22 (95% CI: 1.06–4.64 *p* = 0.035).

**Table 11:** Determinants of elevated triglycerides (≥ 1.7 mmol/L) among women aged 20–49 years in kirinyaga county (*N* = 425).

Variable	Crude OR (95% CI)	*p*-value	Adjusted OR (95% CI)	*p*-value
Age (years)	1.05 (1.01–1.08)	0.007*	1.06 (1.01–1.11)	0.020*
Gravidity	1.04 (0.86–1.26)	0.708	0.79 (0.42–1.45)	0.461
Parity	1.07 (0.87–1.31)	0.524	1.09 (0.58–2.02)	0.797
Years of schooling	0.99 (0.91–1.08)	0.846	1.20 (0.97–1.49)	0.088
Marital status				
Never married	Reference	–	Reference	–
Currently married/Cohabiting	2.15 (0.74–6.25)	0.160	2.20 (0.63–7.72)	0.217
Separated/Divorced/Widowed	3.19 (1.00–10.17)	0.050	2.72 (0.72–10.30)	0.141
Family planning use				
None	Reference	–	Reference	–
Hormonal	0.70 (0.39–1.24)	0.217	0.81 (0.42–1.57)	0.543
Non-hormonal	1.02 (0.20–5.14)	0.979	0.80 (0.14–4.48)	0.798
Intra-Uterine Devices	1.36 (0.60–3.10)	0.460	1.35 (0.56–3.28)	0.503
Education				
Primary incomplete	Reference	–	Reference	–
Primary completed	0.69 (0.32–1.49)	0.346	0.51 (0.21–1.28)	0.151
Secondary incomplete	0.92 (0.38–2.23)	0.859	0.60 (0.16–2.28)	0.452
Secondary completed	0.68 (0.31–1.50)	0.337	0.31 (0.07–1.39)	0.126
College/University	0.63 (0.25–1.60)	0.327	0.22 (0.03–1.59)	0.133
Employment				
Government employee	Reference	–	Reference	–
Non-government employee	0.76 (0.20–2.93)	0.693	0.64 (0.14–2.79)	0.548
Self-employed	0.86 (0.23–3.25)	0.82	0.81 (0.18–3.76)	0.792
Student	0.66 (0.16–2.71)	0.561	0.61 (0.12–3.01)	0.541
Other employment	–	–	–	–
Average income	1.00 (1.00–1.00)	0.696	1.00 (1.00–1.00)	0.286
Wealth quintile				
Low	Reference	–	Reference	–
Middle	1.10 (0.58–2.10)	0.762	1.18 (0.60–2.34)	0.632
High	1.51 (0.81–2.79)	0.194	2.22 (1.06–4.64)	0.035*

OR, odds ratio; CI, confidence interval; AOR, adjusted odds ratio.

* *p* ≤ 0.05.

Table 12 presents the determinants of the metabolic syndrome. The results show that increasing age, gravidity, and parity influenced the metabolic syndrome; age increased the odds of developing the metabolic syndrome at a crude OR of 1.05 (95%CI: 1.02–1.07, *p* = 0.001), and at an adjusted OR of 1.04 (1.01 -1.08, *p* = 0.020). At the same time, gravidity increased the odds of developing metabolic syndrome by 1.16 times, and parity by 1.22 times, with crude ORs of 1.16 (95% CI: 1.00–1.35, *p* = 0.046) and 1.22 (1.04–1.43, *p* = 0.015), respectively. Persons who had completed primary school also had lower odds of developing metabolic syndrome, with a crude OR of 0.54 (0.29–0.99, *p* = 0.048). Among students and persons in informal employment, the odds of developing metabolic syndrome were lower. For students, the crude OR was 0.27 (95% CI: 0.08–0.94, *p* = 0.04) and the adjusted OR was 0.24 (95% CI: 0.06–0.98, *p* = 0.047). For persons in informal employment, the crude OR was 0.13 (95% CI: 0.02–0.76, *p* = 0.024). Persons in the highest wealth quintile were also more likely to develop metabolic syndrome, with an adjusted OR of 1.79 (95% CI: 1.03–3.14, *p* = 0.040).

**Table 12:** Determinants of the metabolic syndrome among women aged 20–49 in kirinyaga county, Kenya.

Variable	Crude OR (95% CI)	*p*-value	Adjusted OR (95% CI)	*p*-value
Age (years)	1.05 (1.02–1.07)	0.001*	1.04 (1.01–1.08)	0.020*
Gravidity	1.16 (1.00–1.35)	0.046*	0.83 (0.56–1.22)	0.429
Parity	1.22 (1.04–1.43)	0.015*	1.30 (0.84–2.01)	0.343
Years of schooling	0.97 (0.91–1.03)	0.36	0.99 (0.84–1.16)	0.885
Marital status				
Never married	Reference	–	Reference	–
Currently married/Cohabiting	1.74 (0.91–3.33)	0.092	1.49 (0.65–3.42)	0.344
Separated/ Divorced/ Widowed	1.61 (0.71–3.63)	0.254	1.11 (0.45–2.77)	0.820
Family planning use				
None	Reference	–	Reference	–
Hormonal	0.68 (0.44–1.06)	0.086	0.67 (0.40–1.13)	0.135
Non-hormonal	1.43 (0.38–5.31)	0.598	1.02 (0.24–4.23)	0.982
Intra-Uterine Devices	1.25 (0.62–2.51)	0.531	0.95 (0.45–2.02)	0.893
Education				
Primary incomplete	Reference	–	Reference	–
Primary completed	0.54 (0.29–0.99)	0.048*	0.61 (0.29–1.28)	0.192
Secondary incomplete	0.85 (0.41–1.74)	0.651	1.45 (0.49–4.31)	0.505
Secondary complete	0.62 (0.33–1.16)	0.136	1.02 (0.30–3.49)	0.979
College/University	0.61 (0.30–1.25)	0.174	0.92 (0.19–4.33)	0.911
Employment				
Government employee	Reference	–	Reference	–
Non-government employee	0.32 (0.10–1.06)	0.063	0.28 (0.07–1.04)	0.057
Self-employed	0.40 (0.12–1.34)	0.139	0.44 (0.11–1.70)	0.233
Student	0.27 (0.08–0.94)	0.04*	0.24 (0.06–0.98)	0.047*
Informal employment	0.13 (0.02–0.76)	0.024*	0.26 (0.04–1.69)	0.156
Average income	1.00 (1.00–1.00)	0.685	1.00 (1.00–1.00)	0.643
Wealth quintile				
Low	Reference	–	Reference	–
Middle	0.88 (0.55–1.41)	0.596	0.92 (0.56–1.53)	0.752
High	1.33 (0.83–2.12)	0.235	1.79 (1.03–3.14)	0.040*

OR, odds ratio; CI, confidence interval; AOR, adjusted odds ratio.

* *p* ≤ 0.05.

## 4 Discussion

This study aimed to determine the prevalence of four metabolic risk factors for noncommunicable diseases, as mapped out by the World Health Organization (1), and the prevalence of metabolic syndrome according to the JIS criteria (2) among women aged 20–49 years in Kirinyaga County, and to examine the sociodemographic determinants associated with these risks.

The prevalence of obesity and overweight in Kirinyaga County was 4.27% higher than the data presented in the 2022 Demographic and Health Survey (11). This study's results reveal that central obesity exceeds obesity by BMI. This trend, in which central obesity exceeds obesity by BMI, has also been observed in Nairobi County, Kenya (24). Central obesity, characterised by the accumulation of visceral fat, is a risk factor for cardiometabolic health, a better predictor of cardiovascular risk and mortality, and a primary contributor to elevated blood glucose, hypertension, and dyslipidemia (25). Biologically, increasing age increases the likelihood of both central obesity and obesity-related BMI, consistent with studies conducted in South Africa, Sub-Saharan Africa and low and middle-income countries (17, 26, 27). Ageing is associated with increased adiposity, as metabolism slows and the beneficial effects of estrogen through its receptors weaken in fat tissue (27). Higher parity and gravidity also increased the likelihood of abdominal obesity and obesity-related BMI, consistent with data from low and middle-income countries (26). Dietary habits and reduced physical activity during the prepartum and postpartum periods often lead to weight gain, which accumulates across pregnancies (28, 29).

Socio-demographically, marital separation increased the prevalence of being obese, agreeing with a study in Mali (30) and differing with studies in Kenya and Tanzania (28, 31), where married women had a higher likelihood of being overweight or obese. While marriage-associated obesity has been linked to a sense of security and peace of mind (32), the high prevalence of obesity among separated women may stem from stress eating. Socio-economically, in Kirinyaga County, informal employment reduced the odds of becoming obese, while in Tanzania, the odds were increased (28). Being in the high and middle-income wealth quintiles also increased the odds of becoming obese, consistent with studies from South Africa and low and middle-income countries (17, 26). The association between increasing wealth and obesity may result from greater capacity to purchase and consume energy-dense foods (28). An increase in years of schooling was also associated with a reduction in abdominal obesity in this study; this was in agreement with a study in Southeastern Turkey (33), where higher levels of education decreased obesity, as measured by BMI, but was contradictory to South Africa and Sub-Saharan Africa, where the prevalence of obesity by BMI is higher in more educated women (17, 27). The reduction in obesity, among women with higher levels of education in this study, might be because education has been shown to increase awareness of personal health, and individuals are more likely to shift to and practice healthier lifestyles (33). Obesity not only predisposes affected individuals to multi-morbidities, but it also has a major economic impact, resulting in losses of 0.8% to 2.4% of the gross domestic product (34). By 2035, 3% of global GDP is projected to be devoted to managing obesity (35).

In Kirinyaga County, 63.8% of the women had elevated fasting glucose, with 55.25% being pre-diabetic and 8.56% being diabetic. In the Kenyan demographic health survey, 1% of the women of reproductive age have been diagnosed with diabetes, and 63% were currently on treatment for diabetes. The observed contrast is likely due to the study's use of more accurate fasting blood glucose measurements, which avoid postprandial fluctuations in random blood glucose measurements (36). Improper and incorrect management of prediabetes usually leads to insulin resistance, which progresses to Type 2 diabetes (37, 38). Type 2 diabetes damages vital organs, including the retina, liver, and kidneys (38, 39). In this study, increasing age increased the odds of having elevated fasting glucose; age-related declines in glucose effectiveness and insulin secretory capacity drive this trend (40). Hormonal contraceptive use was associated with lower odds of elevated blood glucose, consistent with findings in the US (41). However, it was in contrast to studies in Ethiopia (42) and other cohorts where long-term use of hormonal contraceptives (43) or use of specific androgenic progestogens (44) was linked to increased fasting glucose and insulin resistance. The divergent findings might suggest a healthy-user effect, in which women accessing family services benefit from healthcare interactions and screening, which help mitigate metabolic risk factors. Socio-economically, individuals with higher educational status had lower odds of developing elevated blood glucose, consistent with a study in Korea (45). The reduced risk might be due to the positive correlation between educational attainment and health literacy (45). Type 2 diabetes cost Kenya USD 635 million in 2021, with projections reaching USD 1.6 billion by 2045 (46).

The prevalence of elevated blood pressure in our study was 2% higher than that reported in the 2022 DHS data (11). The persistent elevation in arterial blood pressure leads to hypertension, a key risk factor for cardiovascular morbidity and mortality, and a precursor to the metabolic syndrome (47, 48). Consequences of uncontrolled blood pressure include heart failure, heart attacks, strokes, aneurysms, kidney problems, and eye problems (48). In this study, hypertension was associated with increasing age, consistent with studies in Sub-Saharan Africa (49). Age-related changes in the arterial walls and reductions in physical activity are probably the main determinants of this change (50). Gravidity and parity also increased the likelihood of developing hypertension in this study; this aligned with studies from Sudan and Iran (51, 52). Multi-parity increases exposure to various physiological changes, including weight gain, dyslipidemia, insulin resistance, elevated plasma glucose and endothelial dysfunction (16, 52). However, extended breastfeeding may help to mitigate the risks by releasing oxytocin and mobilising lipids (16). In our study, the use of hormonal contraceptive methods reduced the odds of developing hypertension. Systematic reviews indicate that the effects of hormonal contraception vary by composition combinations (53) and delivery methods, with vaginal rings and hormonal intrauterine devices reducing blood pressure, while injectables increase it (53). Socio-demographically, married women also had a higher likelihood of developing hypertension, which agreed with a study in Lesotho, Bangladesh and India (15, 50, 54), possibly due to marital stress. Socio-economically, higher education was associated with reduced odds of hypertension, aligning with data from India (50) but differing from data from Bangladesh (15). In Kenya, the annual cost per patient for hypertension management was USD 304.8, with medications accounting for the largest share at USD 168 (55). This was similar to other studies in sub-Saharan Africa, where hypertension medications accounted for the lion's share of expenditure (56).

Dyslipidemia is responsible for up to 4 million deaths annually and is a major risk factor for ischemic heart disease (8). In this study, dyslipidemia was characterised by a high prevalence of low HDL-C, elevated triglycerides and elevated cholesterol. Compared with the 2015 stepwise survey in Kenya, the prevalence of elevated cholesterol decreased to 13%, while the prevalence of low HDL-C increased to 60% (57). Biologically, increasing age in this study was associated with elevated cholesterol and triglyceride levels, which aligns with studies in Tunisia and China (58, 59). Ageing progressively impairs the absorption, production, and metabolism of fats and lipoproteins (58, 59). Regarding reproductive health, the use of hormonal family planning methods and intrauterine devices was associated with increased odds of having low high-density lipoprotein, which is in agreement with data from Ethiopia and Iran that link these methods to adverse elevations in cholesterol and triglycerides (60–62). Socio-demographically, marital status influenced the lipid profile; separated and divorced women were less likely to have low HDL-C, while women who were previously married had reduced odds of having elevated cholesterol. Marriage is linked to increased triglycerides in Taiwan (63) and dyslipidemia in China (64). Marriage confers a protective effect against cardiovascular disease (65), but this effect depends on relationship stability, as strain erodes its protective benefits (66). Socio-economically, women in the high wealth quintile had higher odds of having elevated triglycerides. This finding mirrors those in Brazil, where high SES correlated with multiple lipid abnormalities (75), but differs from Iran, where women in low SES also had higher triglyceride levels (66).

The prevalence of metabolic syndrome (MetS) in Kirinyaga County, at 46.4%, was significantly higher than the rates reported amongst women of reproductive age in Kenya, Uganda and South Africa (13, 18, 19). MetS elevates the risk of cardiovascular events, and persons with it are five times more likely to develop Type 2 diabetes (13). Age, a natural biological marker, was also associated with a higher odds of metabolic syndrome, consistent with other studies from Kenya and South Africa (13, 18). Ageing has been shown to increase the risk of metabolic disorders due to physiological changes, including reduced metabolic rate, hormonal shifts, and altered body composition (67). Interestingly, the development of metabolic syndrome also accelerates the ageing process in individuals, due to the excess reactive oxygen species present in this condition (68). Increased parity and gravidity in this study were also associated with an increased risk of developing metabolic syndrome, this was in alignment with a meta-analysis that indicated a 12% risk increase per live birth (69), as a result of physiological changes, including weight gain, changes in cholesterol distribution, insulin resistance, and high blood sugar levels (52), that accumulate with multiple pregnancies (69). Socio-economically, completing primary school reduced the odds of developing metabolic syndrome, consistent with data from South Africa (18). This trend suggests that education might be increasing health literacy. Employment and wealth patterns also varied, with students and informal workers having lower odds of developing metabolic syndrome, a finding in agreement with studies in South Africa (18) and China (70). Individuals in the highest wealth quintile were more likely to develop metabolic syndrome in this study, a finding that contrasts with trends in South Africa (18) and China (70). The positive association between wealth and the metabolic syndrome is most likely a reflection of the nutrition transition in developing economies, where higher SES facilitates the consumption of energy-dense, processed foods and sedentary behaviours, unlike in high-income economies, where affluence is typically associated with better health behaviours and lower disease risk (71).

It is critical to acknowledge that this study was cross-sectional and, therefore, presents a snapshot of the picture at a single point in time. It might not provide causality and can only show associations. It was also limited to Kirinyaga County, which is predominantly agricultural. Therefore, it might not be possible to generalise the data to arid or semi-arid regions in Kenya or to highly urbanised regions. The limitations of the study, however, are mitigated by the simultaneous collection of data on both anthropometric and biochemical parameters, which allows for a more precise assessment of individuals’ true metabolic health. The utilisation of fasting blood glucose also helped capture and record participants who would normally be missed when using a random blood sugar measurement. The study's unique focus on a healthy population and the metabolic syndrome also presents an excellent opportunity for interventions that can reverse these risk factors before the development of non-communicable diseases. Consequently, this study fills a significant data gap regarding the epidemiological transition of non-communicable diseases in rural and peri-urban Kenya, providing a solid evidence base for county-level policy formulation.

To reduce the high prevalence of metabolic syndrome and associated risk factors, different policies can be implemented and enforced. At the county level, efforts should focus on increasing the mobilisation of funds for preventive screenings and public health programs to address the nutrition transition. At the national level, Kenya has an NCD strategic plan (72) that outlines multi-sectoral actions to reduce the NCD burden. However, it does not explicitly identify women aged 20–49 as a high-risk group; this should be revised to acknowledge that metabolic health is vital for both maternal survival and the prevention of intergenerational disease transmission. In addition, the social health authority should include an annual, mandatory, and free wellness package for women of reproductive age to facilitate screening for metabolic health. This study points to parity, gravidity, and family planning as key determinants of metabolic risk factors; therefore, prenatal, postnatal, and family planning clinics offer a unique opportunity for the early detection and opportunistic screening for these risk factors and metabolic syndrome. This approach utilises the healthy user effect observed in contraceptive users. The connection between marriage, separation, and adverse metabolic outcomes emphasises the need for gender sensitive interventions that address the psychosocial determinants of health. Policymakers should also invest in qualitative studies to understand the social barriers women face. To sustain these interventions, community health promoters, who are identified as Level 1 providers by the Kenya Community Health Strategy 2020–2025 (73), yet remain under-equipped, should be continuously trained and equipped with functional diagnostic tools to conduct household-level surveillance. This approach enables the early identification of some metabolic risk factors for non-communicable diseases.

## 5 Conclusion

The high prevalence of the metabolic syndrome at 46.4% alongside the high rates of obesity, central adiposity, elevated fasting glucose, elevated blood pressure, and dyslipidemia indicates that women of reproductive age in Kirinyaga County might be at an increased risk of developing non-communicable diseases later in life. Biologically, increasing age was identified as a key risk factor; a look at the reproductive factors shows that multi-parity was also a key factor, which reflects the cumulative impact of reproductive stress on women. When it came to the socio-economic determinants, while the attainment of education offered protection, most likely through improved health literacy, persons in the highest wealth quintiles had an increased risk of both obesity and the metabolic syndrome. This paradox aligns with the nutrition model, which theorises that economic affluence in developing countries facilitates sedentary lifestyles and the consumption of calorie-dense diets. This study diverged from global trends regarding family planning choices and marital status. Hormonal contraceptive use was associated with a favourable glucose and lipid profile, suggesting the prevalence of newer-generation, metabolically neutral formulations in this region or a potential healthy-user effect, in which increased healthcare interactions and screening among women accessing family planning services mitigate the effects of these risk factors. On the other hand, the state of the marital union also had an impact on the metabolic risk factors, where marriage and separation were associated with negative outcomes in domestic and psychosocial stress on women's metabolic health. The findings of this study show the need for public health interventions that go beyond general awareness and address the unique intersection of wealth, parity, and stress in this population.

## Data Availability

The original contributions presented in the study are included in the article/Supplementary Material, further inquiries can be directed to the corresponding author.
