# Supplementary material for: Sociodemographic determinants and prevalence of metabolic risk factors and the metabolic syndrome among women aged 20–49 in kirinyaga county, Kenya
Source: Front Epidemiol. 2026 Aug 6;6:1807836. doi: 10.3389/fepid.2026.1807836 (PMC13490080; doi:10.3389/fepid.2026.1807836)
Supplement: Supplementary file 1 [file Datasheet1.pdf]

SOCIO-DEMOGRAPHIC DETERMINANTS AND PREVALENCE OF METABOLIC RISK FACTORS AND THE METABOLIC SYNDROME AMONG WOMEN AGED 20-49 YEARS IN KIRINYAGA COUNTY, KENYA

Sampling Procedure

A multi-stage random sampling method was employed to select the number of participants in the study. Kirinyaga County has five sub-counties, and one-third of these sub-counties were randomly selected for the study. They include Kirinyaga Central and Kirinyaga North, also known as Mwea West.

Kirinyaga Central and Mwea West have a total of 50,520 women, according to the 2019 Kenya Population and Housing Census. The sample size of 423 persons was thus distributed as follows:

- Kirinyaga Central: (27,181 × 423) ÷ 50,520 = 227.58 ≈ 228
- Kirinyaga North/Mwea West: (23,339 × 423) ÷ 50,520 = 195.42 ≈ 195

The Total number of wards in Kirinyaga Central and Kirinyaga North/Mwea West is 9. One-third of the wards were picked, with one-third from each sub-county. Thus, the study took place in three wards. The intervention stage of the study took place within primary healthcare facilities; therefore, sampling was conducted in community units surrounding these facilities. One-third of all Level 2 and Level 3 healthcare facilities were part of the study. There are 16 Level 2 healthcare facilities, and 5 were selected to participate in the study. Three Level 3 healthcare facilities were also selected from the remaining nine, as per the tables below.

**Sub-County**	**Women**		**Wards**		**Level 2 Facilities**		**Level 3 Facilities**	
	**Total**	**Sample**	**Total**	**Sample**	**Total**	**Sample**	**Total**	**Sample**
Kirinyaga Central	27,181	228	5	2	12	4	3	1
Kirinyaga North/ Mwea West	23,339	195	4	1	4	1	6	2
**Totals**	**50,520**	**423**	**9**	**3**	**16**	**5**	**9**	**3**

Distribution of the sample size across the health facilities that were part of the study:

**Sub-County**	**Wards**	**Level 2 Facilities**	**Level 3 Facilities**

Kirinyaga Central	Ward A: Mutira (n=136)	Facility A: (n=45) Kamuiru Dispensary	Facility A: (n=46) Kagumo Health Centre
		Facility B: (n=45) Gathuthumma Disp	
	Ward B: Kanyekine (n =92)	Facility C (n=46) Gatuto Dispensary	
		Facility D: (n=46) Kiaga Dispensary	
Kirinyaga North/Mwea West	Ward C: Mutithi (n=195)	Facility E: (n=65) Kadongu Dispendary	Facility B: (n=65) Rukanga Health Centre
			Facility C: (n=65) Mutithi Health Centre
