# Supplementary material for: Sociodemographic determinants and prevalence of metabolic risk factors and the metabolic syndrome among women aged 20–49 in kirinyaga county, Kenya
Source: Front Epidemiol. 2026 Aug 6;6:1807836. doi: 10.3389/fepid.2026.1807836 (PMC13490080; doi:10.3389/fepid.2026.1807836)
Supplement: Supplementary file 2 [file Datasheet2.pdf]

SOCIO-DEMOGRAPHIC DETERMINANTS AND PREVALENCE OF METABOLIC RISK FACTORS AMONG WOMEN 20-49 YEARS IN KIRINYAGA COUNTY, KENYA

Supplementary materials: tables mentioned within the text.

Table 1: Socio-demographic characteristics of women aged 20-49 years in Kirinyaga County, Kenya (N=425)

A 100.5% response rate was achieved with 425 participants from Kirinyaga participating in the research study.

Variable	Percentages (%)	Frequency (n)
**Age (years)**		
20–29	23.06	98
30–39	37.88	161
40–49	39.06	166
**Household members under 18**		
Below 4 persons	96.24	409
4 persons and above	3.76	16
**Household members over 18**		
Below 4 persons	87.06	370
4 persons and above	12.94	55
**Marital status**		
Never married	10.82	46
Currently married	71.29	303
Separated	12.24	52
Divorced	2.12	9
Widowed	2.82	12
Cohabitation	0.71	3
**Gravidity**		
≤4 pregnancies	94.35	401
>4 pregnancies	5.65	24
**Parity**		
≤4 births	95.53	406
>4 births	4.47	19
**Family planning use (FP use)**		
None	27.53	117
Non-hormonal	2.35	10
Hormonal	59.76	254
Intrauterine Devices	10.35	44

Categorical variables are reported as frequency (n) and percentage (%)

Table 2. Social Economic Characteristics of women aged 20-49 years in Kirinyaga County (N=425)

Variable	Percentages (%)	frequency (n)
**Highest education**		
No formal schooling	0.94	3
Primary completed	13.18	56
Secondary completed	30.82	131
High school completed	13.88	59
College/University completed	26.82	114
Postgraduate degree	14.35	61
**Employment**		
Government employee	3.29	14
Non-government employee	35.53	151
Self-employed	39.76	169
Student	18.59	79
Informal Employment	2.82	12
**Wealth Quintile**		
Low	33.41	142
Middle	33.65	143
High	32.94	140
**Monthly income (KES)**		
Below 3000	26.35	112
3000 – 7,000	27.53	117
7,000 - 15000	30.36	129
Above 15,000	15.76	67

KES = Kenyan Shilling. Categorical variables are presented as frequency (n) and percentage (%)

Table 3: The prevalence of the metabolic risk factors for non-communicable diseases among women aged 20-49 in Kirinyaga County, Kenya.

Indicator	Percentage (%)	Frequency (n)
**BMI Category n=425**		
Underweight (>18.5kg/m^2)	3.30	14
Normal (18.5kg/m^2 - 24.9kg/m^2)	27.83	118
Overweight (25kg/m^2 -29.9kg/m^2)	29.25	124
Obese (≥ 30kg/m^2)	39.62	168
**Waist Circumference n=425**		
Normal (<80 cm)	20.7	88
Abdominal obesity (≥ 80 cm)	79.3	337
**Waist-Hip Ratio**		
Normal (≤ 0.85)	48.94	208
Abdominal obesity (> 0.85)	51.06	217
**Blood Glucose Levels n=362†**		
Normal (<5.6 mmol/L)	36.19	131
Pre-diabetes (≥5.6mmol/L & ≤ 6.9 mmols)	55.25	200
Diabetic (> 6.9 mmol/L)	8.56	31
**Blood Pressure n=425**		
Normal BP (≤ 130/85)	77.40	329
High BP (≥130/85)	22.60	96
**Dyslipidemia n =425**		
**Triglycerides**		
Normal (<1.7 mmol/L)	82.80	352
Elevated (≥1.7 mmol/L)	17.20	73
**HDL cholesterol**		
Normal (≥1.3 mmol/L)	31.40	124
Low (<1.3 mmol/L)	68.60	271
**Cholesterol**		
Normal (≤5 mmol/L)	92.50	393
Elevated (>5 mmol/L)	7.50	32

†Analysis restricted to participants who fasted (n=362). BMI = Body Mass Index; HDL = High density lipoprotein; BP= blood pressure

Table 4: Prevalence of the Metabolic Syndrome among women aged 20-49 in Kirinyaga County (N=425)

Metabolic Syndrome Status	n	%
No Metabolic Syndrome (< 3 risk factors)	228	53.6
Metabolic Syndrome (≥ 3 risk factors)	197	46.4
Total	425	100

Notes: Metabolic syndrome was defined as the presence of three or more of the following risk factors.

- *a waist circumference greater than or equal to 80 cm,*
- *fasting blood glucose that was greater than or equal to 5.6 mmol/L,*
- *elevated blood pressure, where the systolic blood pressure was greater than or equal to 130mmhg and the diastolic blood pressure was greater than or equal to 85mmhg.*
- *High-density lipoprotein below 1.3 mmol/L and*
- *triglycerides greater than or equal to 1.7 mmol.*

Table 5. Determinants of obesity (BMI ≥ 25kg/m^2) among women aged 20-49 in Kirinyaga County, Kenya (*N=425*).

Variable	Crude OR (95% CI)	p-value	AOR (95% CI)	p-value
**Age (years)**	1.04 (1.01–1.07)	0.003*	1.04 (1.00–1.09)	0.026*
**Gravidity**	1.12 (0.96–1.32)	0.157	0.74 (0.49–1.12)	0.157
**Parity**	1.21 (1.02–1.44)	0.028*	1.37 (0.86–2.17)	0.189
**Years of schooling**	0.98 (0.92–1.06)	0.667	1.08 (0.90–1.29)	0.409
**Marital status**				
Never married	Reference	–	Reference	–
Currently married	1.74 (0.92–3.27)	0.087	0.95 (0.41–2.21)	0.901
Separated	2.56 (1.07–6.12)	0.034*	1.34 (0.48–3.74)	0.576
Divorced	2.69 (0.50–14.39)	0.247	1.54 (0.25–9.56)	0.643
Widowed	1.54 (0.41–5.84)	0.527	0.66 (0.15–2.93)	0.586
Cohabitating	1.54 (0.13–18.19)	0.733	0.59 (0.04–8.99)	0.701
**Family planning use**				
None	Reference	–	Reference	–
Hormonal	1.15 (0.72–1.83)	0.554	1.22 (0.64–3.25)	0.496
Non-hormonal	4.68 (0.57–38.2)	0.150	2.78 (0.32–24.24)	0.355
Intra-Uterine Devices	1.39 (0.66–2.98)	0.404	1.20 (0.52–2.75)	0.668
**Highest education**				
Primary incomplete	Reference	–	Reference	–
Primary completed	1.53 (0.80–2.93)	0.201	1.27 (0.57–2.82)	0.564
Secondary incomplete	2.05 (0.91–4.60)	0.082	1.95 (0.58–6.53)	0.280
Secondary completed	1.16 (0.60–2.23)	0.66	0.85 (0.22–3.24)	0.815
College/University completed	0.96 (0.46–2.00)	0.906	0.55 (0.10–2.91)	0.482
**Employment**				
Government employee	Reference	–	Reference	–
Non-government employee	0.76 (0.20–2.85)	0.681	0.74 (0.18–3.16)	0.704
Self-employed	0.63 (0.17–2.36)	0.494	0.89 (0.20–3.86)	0.880
Student	0.47 (0.12–1.82)	0.275	0.52 (0.11–2.34)	0.395
Informal employment	0.09 (0.01–0.56)	0.01*	0.19 (0.03–1.40)	0.104
**Average income**	1.00 (1.00–1.00)	0.19	1.00 (1.00–1.00)	0.632
**Wealth quintile**				
Low	Reference	–	Reference	–
Middle	1.84 (1.12–3.03)	0.016*	1.84 (1.08–3.13)	0.025*
High	2.07 (1.25–3.44)	0.005*	2.43 (1.33–4.47)	0.004*

Notes: OR*=Odds Ratio;* ***CI****= Confidence Interval;* ***AOR*** *= Adjusted Odds Ratio* ****p ≤ 0.05***

Table 6: Determinants of abdominal obesity (waist circumference ≥80 cm) among women aged 20-49 in Kirinyaga County, Kenya (N=425).

Variable	Crude OR (95% CI)	p-value	AOR (95% CI)	p-value
**Age (years)**	1.03 (1.00–1.05)	0.022*	1.01 (0.97–1.04)	0.704
**Gravidity**	1.29 (1.10–1.50)	0.001*	0.90 (0.61–1.33)	0.554
**Parity**	1.38 (1.17–1.63)	0*	1.49 (0.96–2.31)	0.076
**Years of schooling**	0.94 (0.88–1.00)	0.06*	1.15 (0.97–1.37)	0.106
**Marital status**				
Never married	Reference	–	Reference	–
Currently married	1.57 (0.84–2.94)	0.16	0.82 (0.36–1.83)	0.640
Separated	1.32 (0.59–2.93)	0.501	0.78 (0.31–2.01)	0.614
Divorced	2.84 (0.63–12.80)	0.174	1.81 (0.35–9.37)	0.482
Widowed	1.99 (0.55–7.22)	0.296	0.74 (0.18–3.08)	0.683
Cohabitating	0.71 (0.06–8.41)	0.786	0.30 (0.02–4.66)	0.391
**Family planning use**				
None	Reference	–	Reference	–
Hormonal	1.18 (0.76–1.83)	0.455	1.02 (0.60–1.71)	0.392
Non-hormonal	0.48 (0.12–1.96)	0.309	0.27 (0.06–1.21)	0.088
Intra-Uterine Devices	2.18 (1.06–4.48)	0.034*	1.71 (0.78–3.74)	0.179
**Highest education**				
Primary incomplete	Reference	–	Reference	–
Primary completed	1.33 (0.72–2.47)	0.358	1.09 (0.52–2.30)	0.819
Secondary incomplete	1.82 (0.87–3.82)	0.111	1.27 (0.42–3.87)	0.671
Secondary completed	0.61 (0.32–1.15)	0.124	0.33 (0.09–1.16)	0.085
College/University completed	0.65 (0.32–1.33)	0.240	0.28 (0.06–1.38)	0.118
**Employment**				
Government employee	Reference	–	Reference	–
Non-government employee	0.67 (0.22–2.01)	0.471	0.55 (0.16–1.86)	0.335
Self-employed	0.88 (0.29–2.63)	0.812	0.80 (0.23–2.86)	0.737
Student	0.94 (0.30–2.97)	0.92	0.73 (0.20–2.73)	0.645
Informal employment	0.25 (0.05–1.34)	0.106	0.36 (0.06–2.24)	0.271
**Average income**	1.00 (1.00–1.00)	0.69	1.00 (1.00–1.00)	0.781
**Wealth quintile**				
Low	Reference	–	Reference	–
Middle	1.17 (0.73–1.86)	0.515	1.35 (0.82–2.23)	0.244
High	1.26 (0.79–2.00)	0.34	1.83 (1.04–3.23)	0.036*

***Notes**: **OR**=Odds Ratio; **CI**= Confidence Interval; **AOR** = Adjusted Odds Ratio *p ≤ 0.05*

Table 7. Determinants of elevated blood pressure (BP > 130/85 mmHg) among women aged 20-49 in Kirinyaga County, Kenya (N-425)

Variable	Crude OR (95% CI)	p-value	Adjusted OR (95% CI)	p-value
**Age (years)**	1.10 (1.06–1.14)	<0.001*	1.09 (1.05–1.14)	<0.001*
**Gravidity**	1.28 (1.08–1.53)	0.005*	1.03 (0.66–1.61)	0.884
**Parity**	1.32 (1.09–1.59)	0.004*	0.91 (0.56–1.48)	0.715
**Years of schooling**	0.84 (0.77–0.91)	<0.001*	0.86 (0.73–1.02)	0.078
**Marital status**				
Never married	Reference	–	Reference	–
Currently married/Cohabiting	3.33 (1.16–9.61)	0.026*	1.53 (0.60–7.82)	0.241
Separated/Divorced/Widowed	3.15 (0.94–10.58)	0.063	1.31 (0.34–5.06)	0.696
**Family planning use**				
None	Reference	–	Reference	–
Hormonal	0.34 (0.20–0.56)	<0.001*	0.32 (0.18–0.59)	<0.001*
Non-hormonal	0.79 (0.19–3.24)	0.748	0.49 (0.11–2.26)	0.300
Intra-Uterine Devices	0.78 (0.37–1.65)	0.511	0.54 (0.23–1.24)	0.147
**Education**				
Primary level	Reference	–	Reference	–
Secondary level	0.58 (0.35–0.94)	0.026*	1.53 (0.64–3.65)	0.335
College/University	0.21 (0.08–0.55)	0.002*	0.61 (0.14–2.73)	0.519
**Employment**				
Government employee	Reference	–	Reference	–
Non-government employee	1.07 (0.28–4.04)	0.926	0.54 (0.12–2.44)	0.417
Self-employed	1.17 (0.31–4.41)	0.812	0.57 (0.12–2.63)	0.468
Student	1.01 (0.25–4.02)	0.994	0.50 (0.10–2.47)	0.399
Informal employment	0.33 (0.03–3.72)	0.372	0.73 (0.05–10.30)	0.813
**Average income**	1.00 (1.00–1.00)	0.418	1.00 (1.00–1.00)	0.921
**Wealth quintile**				
Low	Reference	–	Reference	–
Middle	1.11 (0.64–1.93)	0.699	1.35 (0.73–2.50)	0.346
High	0.90 (0.51–1.58)	0.71	1.58 (0.82–3.22)	0.203

***Notes**: **OR**=Odds Ratio; **CI**= Confidence Interval; **AOR** = Adjusted Odds Ratio *p ≤ 0.05*

Table 8. Determinants of elevated fasting blood glucose among women aged 20-49 in Kirinyaga County, Kenya (N=362).

Variable	Crude OR (95% CI)	p-value	Adjusted OR (95% CI)	p-value
**Age (years)**	1.05 (1.03–1.08)	<0.001*	1.03 (0.99–1.07)	0.125
**Gravidity**	1.13 (0.97–1.32)	0.121	0.82 (0.55–1.22)	0.328
**Parity**	1.17 (0.99–1.38)	0.065	1.29 (0.83–2.01)	0.263
**Years of schooling**	0.95 (0.89–1.02)	0.157	1.01 (0.84–1.21)	0.893
**Marital status**				
Never married	Reference	–	Reference	–
Currently married/Cohabiting	1.02 (0.54–1.93)	0.941	1.11 (0.48–2.55)	0.807
Separated/ Divorced/ Widowed	1.96 (0.89–4.35)	0.097	1.37 (0.53–3.56)	0.513
**Family planning use**				
None	Reference	–	Reference	–
Hormonal	0.41 (0.25–0.66)	<0.001*	0.47 (0.27–0.82)	0.009*
Non-hormonal	2.83 (0.34–23.34)	0.333	2.15 (0.25–18.81)	0.489
Intra-Uterine Devices	0.67 (0.341–1.45)	0.311	0.58 (0.26–1.33)	0.200
**Education**				
Primary incomplete	Reference	–	Reference	–
Primary completed	0.68 (0.34–1.36)	0.276	0.73 (0.32–1.67)	0.461
Secondary incomplete	0.32 (0.15–0.70)	0.004*	0.48 (0.15–1.54)	0.218
Secondary complete	0.49 (0.25–0.99)	0.046*	0.64 (0.17–2.44)	0.519
College/University	0.68 (0.31–1.51)	0.346	0.78 (0.15–4.19)	0.777
**Employment**				
Government employee	Reference	–	Reference	–
Non-government employee	0.32 (0.07–1.47)	0.143	0.36 (0.07–1.83)	0.217
Self-employed	0.36 (0.08–1.69)	0.197	0.41 (0.08–2.15)	0.291
Student	0.15 (0.03–0.74)	0.019*	0.20 (0.04–1.07)	0.060
Informal employment	0.17 (0.03–1.09)	0.061	0.25 (0.03–1.91)	0.182
**Average income**	1.00 (1.00–1.00)	0.986	1.00 (1.00–1.00)	0.575
**Wealth quintile**				
Low	Reference	–	Reference	–
Middle	0.64 (0.40–1.04)	0.073	0.65 (0.39–1.10)	0.112
High	0.86 (0.52–1.41)	0.556	1.14 (0.64–2.03)	0.665

***Notes**: **OR**=Odds Ratio; **CI**= Confidence Interval; **AOR** = Adjusted Odds Ratio *p ≤ 0.05*

Table 9. Determinants of Low HDL-Cholesterol (<1.3 mmol/L) among women aged 20-49 in Kirinyaga County (N=425)

Variable	Crude OR (95% CI)	p-value	Adjusted OR (95% CI)	p-value
**Age (years)**	0.97 (0.94–0.99)	0.012*	1.00 (0.96–1.04)	0.955
**Gravidity**	0.90 (0.77–1.06)	0.214	1.08 (0.68–1.72)	0.737
**Parity**	0.88 (0.74–1.05)	0.147	0.84 (0.51–1.39)	0.502
**Years of schooling**	1.04 (0.97–1.12)	0.316	0.95 (0.78–1.16)	0.642
**Marital status**				
Never married	Reference	–	Reference	–
Currently married	0.85 (0.40–1.81)	0.670	0.53 (0.19–1.48)	0.225
Separated	0.26 (0.10–0.66)	0.004*	0.19 (0.06–0.61)	0.005*
Divorced	0.16 (0.03–0.74)	0.019*	0.12 (0.02–0.72)	0.020*
Widowed	0.25 (0.06–1.11)	0.069	0.22 (0.04–1.21)	0.082
Cohabitating	0.63 (0.05–7.64)	0.713	0.22 (0.01–3.22)	0.268
**Family planning use**				
None	Reference	–	Reference	–
Hormonal	2.25 (1.37–6.78)	0.001*	2.08 (1.18–3.65)	0.011*
Non-hormonal	1.92 (0.47–7.85)	0.362	2.78 (0.62–12.52)	0.183
Intra-Uterine Devices	2.75 (1.19–6.36)	0.018*	2.60 (1.33–6.45)	0.039*
**Education**				
Primary incomplete	Reference	–	Reference	–
Primary completed	0.55 (0.28–1.09)	0.085	0.52 (0.22–1.23)	0.136
Secondary incomplete	1.67 (0.70–4.02)	0.250	1.71 (0.46–6.38)	0.425
Secondary complete	1.19 (0.57–2.45)	0.646	1.37 (0.31–5.95)	0.676
College/University	0.74 (0.33–1.65)	0.457	0.75 (0.09–3.64)	0.759
**Employment**				
Government employee	Reference	–	Reference	–
Non-government employee	1.01 (0.32–3.18)	0.988	1.11 (0.31–3.96)	0.871
Self-employed	1.30 (0.41–4.09)	0.653	1.71 (0.45–6.46)	0.429
Student	1.64 (0.49–5.50)	0.425	1.51 (0.38–6.03)	0.556
Other employment	0.97 (0.19–5.03)	0.973	0.58 (0.09–3.64)	0.558
**Average income**	1.00 (1.00–1.00)	0.861	1.00 (1.00–1.00)	0.950
**Wealth quintile**				
Low	Reference	–	Reference	–
Middle	1.06 (0.63–1.76)	0.830	1.07 (0.61–1.87)	0.816
High	1.43 (0.85–2.43)	0.180	1.53 (0.81–2.89)	0.185

***Notes**: **OR**=Odds Ratio; **CI**= Confidence Interval; **AOR** = Adjusted Odds Ratio *p ≤ 0.05*

Table 10. Determinants of elevated total cholesterol (>5.0mmol/L) among women aged 20-49 in Kirinyaga County, Kenya (N=425)

Variable	Crude OR (95% CI)	p-value	Adjusted OR (95% CI)	p-value
**Age (years)**	0.97 (0.94–0.99)	0.012*	1.07 (1.01–1.15)	0.029*
**Gravidity**	0.90 (0.77–1.06)	0.214	0.95 (0.44–2.07)	0.904
**Parity**	0.88 (0.74–1.05)	0.147	0.75 (0.33–1.70)	0.496
**Years of schooling**	1.04 (0.97–1.12)	0.316	0.93 (0.69–1.25)	0.647
**Marital status**				
Never married	Reference	–	Reference	–
Currently married/Cohabiting	0.85 (0.40–1.81)	0.67	6.66 (0.63–70.40)	0.115
Separated/ Divorced/ Widowed	0.26 (0.10–0.66)	0.004*	5.92 (0.52 –66.75)	0.151
**Family planning use**				
None	Reference	–	Reference	–
Hormonal	0.47 (0.21–1.07)	0.072	0.59 (1.33–7.47)	0.275
Non-hormonal	–	–	–	–
Intra-Uterine Devices	1.66 (0.61–4.52)	0.325	1.79 (0.57–5.57)	0.316
**Education**				
Primary incomplete	Reference	–	Reference	–
Primary completed	0.55 (0.28–1.09)	0.085	0.88 (0.22–3.52)	0.144
Secondary incomplete	1.67 (0.70–4.02)	0.25	2.42 (0.34–17.15)	0.408
Secondary complete	1.19 (0.57–2.45)	0.646	2.84 (0.31–26.24)	0.676
College/University	0.74 (0.33–1.65)	0.457	2.15 (0.12–36.58)	0.595
**Employment**				
Government employee	Reference	–	Reference	–
Non-government employee	1.01 (0.32–3.18)	0.988	0.15 (0.01–1.87)	0.140
Self-employed	1.30 (0.41–4.09)	0.653	0.33 (0.03–3.90)	0.382
Student	1.64 (0.49–5.50)	0.425	0.26 (0.02–3.53)	0.313
Other employment	0.97 (0.19–5.03)	0.973	–	–
**Average income**	1.00 (1.00–1.00)	0.861	1.00 (1.00–1.00)	0.920
**Wealth quintile**				
Low	Reference	–	Reference	–
Middle	1.06 (0.63–1.76)	0.83	1.13 (0.45–2.83)	0.798
High	1.43 (0.85–2.43)	0.18	1.13 (0.38–3.33)	0.829

***Notes**: **OR**=Odds Ratio; **CI**= Confidence Interval; **AOR** = Adjusted Odds Ratio *$p \leq 0.05$*

Table 11: Determinants of elevated triglycerides (≥ 1.7 mmol/L) among women aged 20-49 years in Kirinyaga County (N=425)

Variable	Crude OR (95% CI)	p-value	Adjusted OR (95% CI)	p-value
**Age (years)**	1.05 (1.01–1.08)	0.007*	1.06 (1.01–1.11)	0.020*
**Gravidity**	1.04 (0.86–1.26)	0.708	0.79 (0.42–1.45)	0.461
**Parity**	1.07 (0.87–1.31)	0.524	1.09 (0.58–2.02)	0.797
**Years of schooling**	0.99 (0.91–1.08)	0.846	1.20 (0.97–1.49)	0.088
**Marital status**				
Never married	Reference	–	Reference	–
Currently married/Cohabiting	2.15 (0.74–6.25)	0.160	2.20 (0.63–7.72)	0.217
Separated/Divorced/Widowed	3.19 (1.00–10.17)	0.050	2.72 (0.72–10.30)	0.141
**Family planning use**				
None	Reference	–	Reference	–
Hormonal	0.70 (0.39–1.24)	0.217	0.81 (0.42–1.57)	0.543
Non-hormonal	1.02 (0.20–5.14)	0.979	0.80 (0.14–4.48)	0.798
Intra-Uterine Devices	1.36 (0.60–3.10)	0.460	1.35 (0.56–3.28)	0.503
**Education**				
Primary incomplete	Reference	–	Reference	–
Primary completed	0.69 (0.32–1.49)	0.346	0.51 (0.21–1.28)	0.151
Secondary incomplete	0.92 (0.38–2.23)	0.859	0.60 (0.16–2.28)	0.452
Secondary completed	0.68 (0.31–1.50)	0.337	0.31 (0.07–1.39)	0.126
College/University	0.63 (0.25–1.60)	0.327	0.22 (0.03–1.59)	0.133
**Employment**				
Government employee	Reference	–	Reference	–
Non-government employee	0.76 (0.20–2.93)	0.693	0.64 (0.14–2.79)	0.548
Self-employed	0.86 (0.23–3.25)	0.82	0.81 (0.18–3.76)	0.792
Student	0.66 (0.16–2.71)	0.561	0.61 (0.12–3.01)	0.541
Other employment	–	–	–	–
**Average income**	1.00 (1.00–1.00)	0.696	1.00 (1.00–1.00)	0.286
**Wealth quintile**				
Low	Reference	–	Reference	–
Middle	1.10 (0.58–2.10)	0.762	1.18 (0.60–2.34)	0.632
High	1.51 (0.81–2.79)	0.194	2.22 (1.06–4.64)	0.035*

***Notes**: **OR**=Odds Ratio; **CI**= Confidence Interval; **AOR** = Adjusted Odds Ratio *p ≤ 0.05*

Table 12. Determinants of the metabolic syndrome among women aged 20-49 in Kirinyaga County, Kenya

Variable	Crude OR (95% CI)	p-value	Adjusted OR (95% CI)	p-value
**Age (years)**	1.05 (1.02–1.07)	0.001*	1.04 (1.01–1.08)	0.020*
**Gravidity**	1.16 (1.00–1.35)	0.046*	0.83 (0.56–1.22)	0.429
**Parity**	1.22 (1.04–1.43)	0.015*	1.30 (0.84–2.01)	0.343
**Years of schooling**	0.97 (0.91–1.03)	0.36	0.99 (0.84–1.16)	0.885
**Marital status**				
Never married	Reference	–	Reference	–
Currently married/Cohabiting	1.74 (0.91–3.33)	0.092	1.49 (0.65–3.42)	0.344
Separated/ Divorced/ Widowed	1.61 (0.71–3.63)	0.254	1.11 (0.45–2.77)	0.820
**Family planning use**				
None	Reference	–	Reference	–
Hormonal	0.68 (0.44–1.06)	0.086	0.67 (0.40–1.13)	0.135
Non-hormonal	1.43 (0.38–5.31)	0.598	1.02 (0.24–4.23)	0.982
Intra-Uterine Devices	1.25 (0.62–2.51)	0.531	0.95 (0.45–2.02)	0.893
**Education**				
Primary incomplete	Reference	–	Reference	–
Primary completed	0.54 (0.29–0.99)	0.048*	0.61 (0.29–1.28)	0.192
Secondary incomplete	0.85 (0.41–1.74)	0.651	1.45 (0.49–4.31)	0.505
Secondary complete	0.62 (0.33–1.16)	0.136	1.02 (0.30–3.49)	0.979
College/University	0.61 (0.30–1.25)	0.174	0.92 (0.19–4.33)	0.911
**Employment**				
Government employee	Reference	–	Reference	–
Non-government employee	0.32 (0.10–1.06)	0.063	0.28 (0.07–1.04)	0.057
Self-employed	0.40 (0.12–1.34)	0.139	0.44 (0.11–1.70)	0.233
Student	0.27 (0.08–0.94)	0.04*	0.24 (0.06–0.98)	0.047*
Informal employment	0.13 (0.02–0.76)	0.024*	0.26 (0.04–1.69)	0.156
**Average income**	1.00 (1.00–1.00)	0.685	1.00 (1.00–1.00)	0.643
**Wealth quintile**				
Low	Reference	–	Reference	–
Middle	0.88 (0.55–1.41)	0.596	0.92 (0.56–1.53)	0.752
High	1.33 (0.83–2.12)	0.235	1.79 (1.03–3.14)	0.040*

***Notes**: **OR**=Odds Ratio; **CI**= Confidence Interval; **AOR** = Adjusted Odds Ratio *$p \leq 0.05$*
